# Cell cycle regulators and bone: development and regeneration

**DOI:** 10.1186/s13578-023-00988-7

**Published:** 2023-02-21

**Authors:** Alisha Shaikh, Austin A. Wesner, Mohanad Abuhattab, Raman G. Kutty, Priyatha Premnath

**Affiliations:** 1 Department of Biomedical Engineering, University of Wisconsin-Milwaukee, College of Engineering and Applied Sciences, 3200 N Cramer St, Milwaukee, WI 53211 USA; 2 Department of Internal Medicine, White River Health System, Batesville, AR USA

**Keywords:** p21, Cell cycle, Regeneration, Bone

## Abstract

Cell cycle regulators act as inhibitors or activators to prevent cancerogenesis. It has also been established that they can play an active role in differentiation, apoptosis, senescence, and other cell processes. Emerging evidence has demonstrated a role for cell cycle regulators in bone healing/development cascade. We demonstrated that deletion of p21, a cell cycle regulator acting at the G1/S transition enhanced bone repair capacity after a burr-hole injury in the proximal tibia of mice. Similarly, another study has shown that inhibition of p27 can increase bone mineral density and bone formation. Here, we provide a concise review of cell cycle regulators that influence cells like osteoblasts, osteoclasts, and chondrocytes, during development and/or healing of bone. It is imperative to understand the regulatory processes that govern cell cycle during bone healing and development as this will pave the way to develop novel therapies to improve bone healing after injury in instances of aged or osteoporotic fractures.

## Introduction

Bone healing and development are regulated by a myriad of factors including interactions with the extracellular matrix, other cells, and intracellular factors. One such factor, cell cycle, is frequently considered to aid in proliferation. However, studies indicate that cell cycle or exit of cell cycle also plays a vital role in differentiation, senescence, and quiescence. Bedelbaeva et al. were the first to illustrate a firm link between loss of p21 and appendage regeneration [1]. Subsequently, other studies determined a link between p21 and regeneration of other tissues such as liver [2], cartilage [3] and heart [4]. Recently, we demonstrated that lack of p21 can increase bone formation after an injury, both bone volume and bone mineral density [5]. Another regulator that has displayed an association to bone and its processes is p27 [6, 7]; where it has been shown to increase osteoblast differentiation and mineralization. While not directly, several other cell cycle regulators have also demonstrated their influence on bone related mechanisms.

## Cell cycle and bone

### Cell cycle

**Fig. 1:**
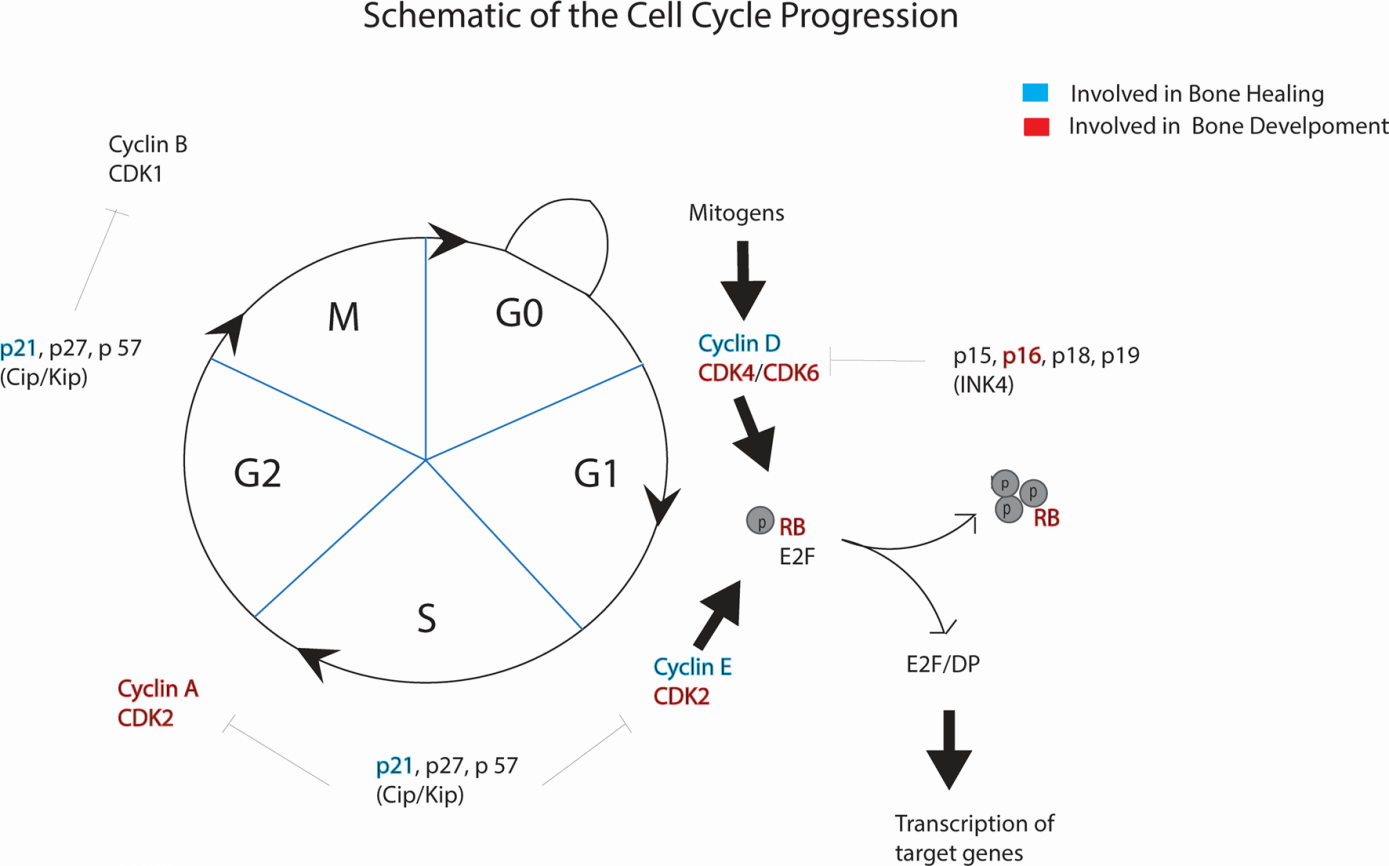
Cell cycle and its constituent regulators [12]. Regulators that take part in bone development and bone healing are separately highlighted (blue for bone healing and red for bone development). Adapted with permission (Volume: 1866, Issue: 5, Pages: 1–10. ^©^2020 Elsevier B.V.)

In eukaryotes the cellular cycle is a well-concerted process that regulates cellular division wherein one mother cell gives rise to two identical daughter cells. Broadly, the mitotic cycle consists of two phases, interphase and M phase. Interphase prepares the cell for cell division; the cell grows and duplicates its genetic material while the physical separation of the two daughter cells takes place in M phase [8]. Interphase is subdivided into three phases, G_1_ (first gap phase), S (synthesis), and G_2_ (second gap phase). RNA and protein are synthesized throughout G_1_, S, and G_2_. As each daughter cell must be functional at the end of cellular division, this synthetic process serves both the purposes of producing the machinery necessary to undergo cellular division as well as providing the daughter cells with functioning organelles [9]. Although this protein and RNA synthesis occurs throughout interphase, DNA is duplicated only in S phase [10]. During G_2_ the cell also grows in size, effectively doubling its cytoplasm [11]. Once the cell has the necessary components for division, i.e., DNA, RNA, protein, organelles, and cytoplasm, the cell enters the M phase. In M phase, chromosomes are pulled to the poles of the mother cell and organelles are segregated equally and once completed, the cell membrane cleaves, and two complete daughter cells are formed. The cell cycle, its constituent regulators and their role in healing and development are summarized in Fig. 1.

In order to undergo cellular division, the cell must exert a tremendous amount of energy. Accordingly, it is critical that the cell have mechanisms in place to ensure absolute fidelity in the process as well as govern the sequence by which the steps necessary for division are executed. This strict regulation is made possible by checkpoint proteins [13], the functions of which in regards to bone healing and development will be the focus of this review.

### Mechanisms of bone healing and development

Prior to discussing the role of cell cycle in healing, we will first elaborate on the process of bone healing and development. During development, osteogenesis occurs where preexisting mesenchymal tissue is transformed into bone tissue that is derived from the paraxial mesoderm [14]. Bone tissue is composed of four different cell types: osteoprogenitor cells, osteoblasts, osteocytes, and osteoclasts. Bone contains a small number of osteoprogenitor cells that differentiate from mesenchymal stem cells (MSCs) found in the bone marrow which can multiply and differentiate into osteoblasts [15, 16]. Osteoblasts also differentiate from periosteal cells during fracture healing [17]. Osteoblasts mature into osteocytes when surrounded by a bone matrix [15], maintaining bone function acting as mechanosensors [18]. Finally, osteoclasts are bone reabsorbing cells that enable bone remodeling in homeostasis and bone healing through the production of proteolytic enzymes and secretion of hydrogen ions [19]. In addition to bone cells, cartilage cells or chondrocytes are also significant in the formation of bone. Emerging evidence points to some hypertrophic chondrocytes transdifferentiating into osteoblasts during endochondral ossification rather than only undergoing apoptosis and subsequently being replaced by osteoprogenitor cells that differentiate into osteoblasts [20]. The bone and cartilage cells together take part in the development and regeneration of bone.

There are two distinct ways in which bone can form; intramembranous and endochondral ossification [21]. Endochondral ossification is the primary method for bone formation in the body. Here, MSCs differentiate into chondrocytes and form cartilage models of future bones. In the first phase of endochondral ossification, the perichondrium is vascularized [22], resulting in blood vessels starting to deliver nutrients that stimulate the MSCs to differentiate into osteoblasts [23]. In the second phase, these newly formed osteoblasts will gather at the diaphysis to form a bone collar, stimulating bone regeneration. Chondrocytes that remain in the center of the bone will hypertrophy and signal the surrounding cartilage to calcify [24]. The calcification matrix will result in impermeability toward the inner portion of the developing bone, causing cell death. The periosteal bud is then formed which invades the cavity resulting in the formation of spongy bone. The periosteal bud consists of arteries, veins, lymphatic vessels and nerves, which participate in delivering osteogenic cells. In this phase, osteoclasts degrade the cartilage matrix while osteoblasts deposit new spongy bone. As mentioned previously, not all chondrocytes undergo apoptosis after hypertrophy but some transdifferentiate to a stromal cell-like morphology followed by differentiation to osteoblasts [25].

On the other hand, during intramembranous ossification MSCs differentiate directly into osteoblasts to form bone. Osteoblasts initiate the secretion of osteoid, which is responsible for the hardness of the bone. In the second step of intramembranous ossification, peripheral MSCs continue to differentiate into osteoblasts [26]. Osteoblasts secrete matrix proteins such as osteocalcin and osteopontin, and transports minerals into the matrix that occurs toward the ossification center. As the secretion continues inward, the osteoblast becomes trapped within the ossification center and eventually differentiates further to become an osteocyte. The osteoid calcifies and hardens over time, resulting in the formation of the bone matrix [27]. The osteoblast continues depositing the osteoid but in a random manner around the blood vessels. This random deposit will finally form a finely woven trabecula. The last step of intramembranous ossification is when the lamellar bone starts to form toward the outer edge of the trabecular bone into a layered structure [28].

The process of bone healing after a fracture can occur directly (primary) or indirectly (secondary). Direct healing occurs with surgery and demobilization using compression plates and screws to stabilize the bone with minimal interfragmentary motion. It involves remodeling of lamellar bone, osteons, and blood vessels. In this pathway, the bone is formed through intramembranous ossification. However, in indirect fracture healing, fractures are stabilized using casts and braces that allow some interfragmentary motion; this is the most common mechanism of bone healing. This path involves the combination of endochondral and intramembranous bone healing, which is enhanced by some interfragmentary motion and weight applied on the bone [29].

When fracture of the bone takes place, an inflammatory response immediately occurs. During the fracture, the blood vessels that supply blood to the bone and periosteum rupture causing a hematoma at the fracture ends, forming a template for callus formation. The inflammatory response, which peaks at 24 h after injury, involves secretion of pro-inflammatory cytokines like tumor necrosis factor and several interleukins [29]. These cytokines attract macrophages that remove damaged tissue and secrete vascular endothelial growth factor (VEGF) which leads to angiogenesis [30]. In response to the inflammation at the fracture site, the majority of MSCs are recruited locally from the inner cellular layer of the periosteum and from the bone marrow. However, they are also recruited from circulating blood [29, 31]. These MSCs then differentiate into fibroblasts, chondroblasts, and osteoblasts to initiate the bone formation phase of healing. A collagen-rich fibrocartilaginous network starts to span the fracture ends: chondrogenesis [30].

After the initial inflammation phase, in 5–11 days, a soft callus due to endochondral ossification forms external to the periosteum. At the same time, intramembranous ossification occurs subperiosteally at the ends of the fracture, generating a hard callus [32]. The soft cartilaginous callus is resorbed to form a hard, bony callus, similar to embryonic bone formation. Calcium granules are transported into the extracellular matrix by means of mitochondria, and are precipitated, along with phosphate, to form mineral deposits, after 14 days. Calcified cartilage is then replaced with woven bone, and the callus becomes more solid [31]. The remodeling process is then carried out leading to hard callus resorption by osteoclasts and lamellar bone deposition by osteoblasts. This process is initiated at 3–4 weeks and takes months-years to complete and requires adequate blood supply and a gradual increase of mechanical stability [29].

## Regulatory control of the cellular cycle in bone

Numerous in vitro and in vivo studies have investigated the involvement of factors that are crucial for bone formation or differentiation of cells. One of those factors include the cell cycle and its regulators. As discussed in the previous section, cell cycle is a highly complex process that regulates the growth and proliferation of cells, regulation of DNA damage repair, tissue hyperplasia due to an injury, and more [8]. It is a series of events in which the components of the cells are doubled and accurately segregated into daughter cells [33]. The cell cycle regulators are thought to influence the differentiation of cells, as withdrawal from the cell cycle or a temporal arrest in the G1 phase is believed to be a requirement for cell differentiation [34]. Given the constant turnover of cellular components in bone, regulation of the cell cycle is of critical importance in bone development and bone remodeling. Here, we provide a concise summary of the cell cycle regulators that have been reported to control the differentiation of osteoblasts, osteoclasts, chondrocytes, and other types of cells or are currently being studied as a target for bone regeneration or development. Cell cycle regulators that have not been reported to have a role in bone healing or development may not be included here.

### Cell cycle regulators

The regulation of cell cycle is essential for the survival of the cell, including the detection and repair of genetic damage as well as preventing uncontrolled cell division. The cell cycle is regulated primarily by Cyclin-dependent kinases (CDKs) and Cyclins in complex. CDKs are serine/threonine kinases whose catalytic activities are modulated by interactions with Cyclins and CDK inhibitors (CKIs) [35]. The interaction between CDKs, Cyclin, and CKI is essential for ensuring a systematic progression through the cell cycle. They also play an important role in transcription, metabolism, neural function, and stem cell self-renewal [35]. In vitro experiments as well as genetically engineered mouse models have demonstrated an intricate role for cell cycle regulators in bone and its processes. The following sections will discuss various cell cycle regulators in the context of bone development, healing, and regeneration.

#### Cyclin-dependent kinases

CDKs depend on their association with a noncatalytic regulatory subunit called a cyclin. CDKs drive the major cell cycle transition points (G1, S, G2, M). While CDK protein levels remain stable throughout the cell cycle, cyclin levels fluctuate causing periodic activation of CDKs. Progression through each phase of the cell cycle requires different CDK-cyclin pairs. CDKs exert control of eukaryotic cell division by regulating cell-cycle stages through the phosphorylation of various substrates. The CDKs present in humans include CDK1, CDK2, CDK3, CDK4, CDK5, CDK6, CDK7, CDK8; however, only some cell cycle regulators have been shown to interact with cells that play a role in bone processes.

##### CDK2

CDK2 is a serine/threonine protein kinase that controls the G1/S transition in the cell cycle, regulates the exit from S phase, promotes DNA replication, and has been found to promote the G2/M DNA damage response checkpoint [36]. In the G1/S phase, CDK4 and CDK6 complex with cyclin D and initially phosphorylate the retinoblastoma (Rb) protein which is crucial for preventing excessive cell growth. The association of CDK2 with cyclin E then completes the phosphorylation of Rb. CDK2 also complexes with cyclin A to control the transition from S to G2 in the cell cycle. In addition, p21(Cip1) and p27(Kip1), which belong to the Cip/Kip protein family, can form a complex to block CDK2/cyclin E and CDK2/cyclin A kinase activity. Studies have shown that CDK2 is dispensable for cell proliferation [37] and mouse development [38]. In the absence of CDK2, CDK1 can phosphorylate Rb by binding to D-type cyclins and can promote replication as a complex with cyclin E1 and Cyclin A [39]. Subsequent inactivation of the cyclin E-CDK2 complex along with the induction of p21 and activation of Rb influences the fibroblast growth factor (FGF), which is thought to be a negative regulator of chondrocyte growth [40].

##### CDK4

CDK4 is a catalytic subunit of the protein kinase complex which plays a role in the regulation of the G1-S transition of the cell cycle. It forms molecular complexes with the members of the D-type cyclin family and is responsible for the phosphorylation of Rb. The activity of CDK4 is negatively inhibited by the CDK inhibitor p16 (INK4a). Additionally, the overexpression/amplification of CDK4 is associated with tumorigenesis of a variety of cancers [41]. Activation of CDK4 phosphorylates the Rb family of proteins, resulting in negative regulation of the passage of cells from G1 to S phase by sequestering transcription factors critical for G1/S transition. The main outcome of CDK4 activation is the inhibition of Rb leading to G1-S cell-cycle transition. CDK4 also directly phosphorylates other proteins which promote cell-cycle progression and inhibit both cell senescence and apoptosis [42]. Studies on the influence of CDK4 in other bone related cells have not been reported. Abella et al. reported that CDK4 is a key regulator of adipocyte differentiation, one of the cell types that an MSC can differentiate into [43].

##### CDK6

CDK6 is important for G1 phase progression and G1 to S phase transition of the cell cycle. The activity of this kinase, which initially appears in the middle of G1 phase, is controlled by D-type cyclins and members of the INK4 family of CDK inhibitors (p16, p15, p18, p19). In addition, this kinase has been shown to phosphorylate and regulate the activity of the tumor suppressor protein Rb [44]. Studies have shown that CDK4 and CDK6 in conjunction with cyclin D1 enhances Rb phosphorylation and its related proteins p107 and p130 in the G1 phase of the cell cycle [45]. In vitro studies have shown that CDK6 is one of the key regulators in the differentiation of multiple types of cells [34]. Its downregulation is critical in controlling osteoblasts, osteoclasts, and chondrocyte differentiation [34]. However, the mechanism by which CDK6 controls cell differentiation without influencing the cell cycle is still unknown. Therefore, the possibility of CDK6 being a target in bone regeneration remains to be seen.

#### Cyclin-dependent kinase inhibitors

Cyclin-dependent kinase inhibitors (CKIs) restrain CDK activity [35]. CKIs are divided into two classes based on their structure and CDK specificity. The INK4 family members—p16, p15, and p19 that primarily target CDK4 and CDK6, and the Cip/Kip family members—p21, p27, and p57 that interfere with the activities of Cyclin A-, D-, and E-dependent kinase complexes.

##### INK4

***p15, p18: Mice*** lacking p15 p19 were reported to have similar phenotype compared to wildtype mice. This suggests that their role in bone development could be compensated for by other cell cycle regulators [71–73]. p18 knockout mice were born with similar characteristics to their wildtype counterparts; however, over a period of 3 weeks they became distinctly larger. Mechanisms behind this increase in weight is still unknown and cannot completely be attributed inhibition of p18 alone [74].

***p16:*** p16 (INK4a or CDK2NA) is an important CKI and a tumor suppressor gene that is not only required for the control of unregulated cell growth in most cell types, but also has roles in other cell cycle phases. Its typical role is to check the cell cycle in the early G1 phase and inhibit further transition of the cell cycle from G1 to S phase. p16 binds to CDK4 and inhibits its interaction with cyclin D causing prevention of passage through the G1 phase of the cell cycle [46].The induction of p16 results in a G1 cell cycle arrest by inhibiting phosphorylation of the Rb protein by the cyclin-dependent kinases CDK4 and CDK6 and may also cause inhibition of CDK2 activity [47]. p16 has been reported to play an important role in cell differentiation, cell quiescence, and cell senescence, which makes it a crucial cell regulatory protein for the regulation of terminal differentiation and the aging process [48]. Therefore, further investigation on p16 seems very promising. There have been no studies linking p16 to any bone processes. However, since p16 plays a role in differentiation it is plausible that it could play a role in MSC differentiation as well.

***p19:*** p19 (INK4d) interacts with CDK4 and CDK6 inhibiting them from binding with cyclin D, resulting in the arrest of the cell cycle in the G0/G1 phase. p19 was found to be present at low levels at the onset of G0/G1 and then accumulates at the entry to S phase and remains elevated through S phase into G2 [49]. Induction of this gene was found to contribute to cell cycle arrest, and knockdown of the gene alone found cells to be sensitive to autophagic cell death. p19 is induced to inhibit the proliferation of many kinds of tumor cells like T cell acute lymphoblast leukemia cells. It is also involved in hematopoietic stem cell quiescence and megakaryocyte/granulocytic differentiation which is associated with cell cycle arrest [50]. Like previous INK4 candidates, p19 also has not been reported to directly take part in bone processes. Yet, its involvement in hematopoietic stem cell and inflammatory cell differentiation could contribute to bone healing and regeneration after an injury and is worth exploring.

##### Cip/Kip

***p21***: p21, also known as CIP1 and Waf1, is a CKI that has been involved in cell differentiation, apoptosis and cell proliferation—its inhibition leads to enhanced proliferation of cells. It binds to cyclin-dependent kinase (CDK) 2 and 4 and inhibits its activity. It functions as a regulator of cell cycle progression at the G1 phase. Expression of this gene is controlled by p53 and this protein plays a regulatory role in S phase DNA replication and repair [51]. p21 expression can also be regulated independently of p53 in certain instances like tissues during development and in the adult mouse [52].

Our lab has demonstrated that p21KO mice displayed enhanced bone regeneration capacity after a burr-hole injury in the proximal tibiae measured over 4 weeks [5]. Our results indicate that MSC numbers in bone marrow were not different between the two mouse types, yet, at the site of injury there were significantly more MSCs after 1 week. The osteogenic differentiation capacity of both mice was investigated, and no significant differences were observed. We hypothesize that the increased number of MSCs at the site of injury either play a direct role by enhancing chondrogenesis or an indirect role by expressing trophic factors. Our lab is currently testing this hypothesis by upregulating a downstream effector, E2F1 specifically in chondrocytes. Preliminary results indicate that mice where E2F1 is overexpressed in chondrocytes exhibit enhanced bone healing albeit with reduced bone mineral density [53]. Of all cell cycle regulators, p21 has demonstrated the most promise to be successfully embedded in interventional therapies for bone healing and regeneration. Of significance is also our finding where inhibition of p21 has shown to protect against bone loss in an osteoporotic environment, potentially by overcoming the absence of estrogen; estrogen and p21 have redundant interactions with osteoclasts [54].

***p27***: p27, known as KIP1, is a CKI that prevents the activation of cyclin E or D, thus controlling cell cycle progression at the G1 phase. The degradation of the protein, triggered by CDK dependent phosphorylation, is required for cell transition from quiescence to the state of proliferation [55]. p27 is an atypical tumor suppressor which regulates G0 to S phase by binding and regulating CDK2, CDK4, and CDK6. In the early G1 phase, p27 translation is at its highest point causing binding and inhibition of cyclin E. Decrease of p27 throughout the G1 phase allows cyclin E and cyclin A to activate gene transcription required for G1-S transition [55, 56]. The p27 pathway has been shown to modulate skeletal growth through bone formation by interacting with the parathyroid hormone-related peptide (Pthrp). When p27 was deleted in Pthrp KI mice, skeletal growth retardation and defective osteoblastic bone formation was rescued [7]. Another study by Yin et al. demonstrated that p27 negatively regulates alveolar bone development, in agreement with the previously described study [57]. p27 plays a role in bone and bone cells and also in osteoprogenitor cells. Bone marrow osteoprogenitor cells from p27KO mice exhibited increased proliferative capacity and formed larger osteoblastic colonies while also differentiating to the mineralization stage [6]. From these studies it is evident that p27 might also be worth considering as a potential target to improve bone related processes like healing and regeneration.

***p57:*** p57 (Kip2) is a strong inhibitor of the G1 cell-cycle CDK complexes. p57 was found to be a negative regulator of cell proliferation [58]. The kinases p21, p27, and p57 have an affinity to bind to, and inhibit, the CDK 2, 4, and 6 [59]. The over expression of p57 leads to cell-cycle arrest in the G1 phase [60]. p57 has also shown that it influences osteoblasts as deletion of p57 can upregulate osteoblasts proliferation and differentiation while also improving bone mineral density [61, 62].

#### Cell cycle cyclins

Cyclins function as regulators of CDKs. Cyclins drive the events of the cell cycle by coupling with CDKs to activate them, making it a functional enzyme allowing it to modify target proteins. Different cyclins exhibit distinct expression and degradation patterns which contribute to coordination of each mitotic event.

***Cyclin A:*** Cyclin A can activate two different CDKs (CKD1 and CDK2), making it particularly interesting among the cyclin family. Cyclin A was also found to bind to the Rb gene family, E2F1 and p21 [63, 64]. Cyclin A is needed in the S and the G2 phase of the cell cycle and reaches a maximal level right before mitosis, after which it degrades rapidly. The only study connecting cyclin A with bone processes is through MSCs, where Fei et al., demonstrated that osteoprotegerin (OPG) deficient mice showed the osteogenic growth peptide (OGP) stimulated MSC proliferation and increased the expression of Cyclin A and CDK2 at mRNA and protein levels [65]. OPG triggered the Cyclin A-CDK2 pathway, resulting in the proliferation of MSCs of OPG-deficient mice. It is however unknown if the MSCs that proliferated also differentiated into bone cells.

***Cyclin D1:*** Cyclin D1 forms a complex with, and functions as a regulatory subunit of, CDK4 and CDK6. In the G1 phase of the cell cycle, Cyclin D1 and its CDK partner are responsible for transition into the S phase through phosphorylation of the Rb gene which will then cause the release of transcription factors for the initiation of DNA replication [64]. Cyclin D1 has been shown to interact with the Rb gene family, where the expression of Cyclin D1 is regulated positively by Rb. Cells that lack functional Rb have significantly lower amounts of Cyclin D1 and Cyclin D1-CDK4 complexes, thus exhibiting a negative feedback loop in which Cyclin D1 synthesis and activation leads to Rb phosphorylation, which then decreases Cyclin D1 expression. In mice lacking cyclin D1, a small skeletal phenotype was observed along with a 50% chance of malformation of the jaw caused by misalignment of the incisor [66]. However, it should be noted that the dwarfism phenotype observed could be a result of lower levels of growth hormones or pituitary issues rather than bone development.

***Cyclin D2***: Cyclin D2, like Cyclin D1 forms a complex with CDK4 and CDK6. CDK4 and CDK6 are associated with the D-type Cyclins during the G1 phase of the cell cycle. Cyclin D2 reaches its maximum activity during the G1 phase and regulates transition into S phase through phosphorylation of the Rb gene [64]. Cyclin D2 inactivates Rb by phosphorylation and induces the release of E2F [67]. In human MSCs, the overexpression of Cyclin D2 promoted proliferation of the cells. Cyclin 2 could be considered a target for increasing MSC numbers, but like with cyclin A, converting these MSCs to bone cells still requires investigation.

#### Retinoblastoma (Rb)

The protein encoded by Rb is a negative regulator of cell cycle. It was found to stabilize heterochromatin to maintain the overall chromatin structure. Hypo-phosphorylated forms of this protein bind to the transcription factor E2F1, meaning through under-phosphorylation of Rb, the G1 cell cycle begins to function in an antiproliferative stage [40, 64]. The Rb gene and its relatives p107 and p130 encode proteins which share several properties including one which inhibits cell-cycle progression [68]. Rb plays a role in maintaining quiescence in adult stem cells, when needed Rb is transiently inactivated to allow for self-renewal and differentiation of stem cells [69]. In addition to cell cycle, Rb plays a vital role in cell adhesion. Sosa-Garcia et al., demonstrated that when Rb is inhibited osteoblasts do not form cell–cell contacts but do continue to proliferate [70]. While proliferation of osteoblasts is important in bone development and healing, loss of cell adhesion can result in metastasis rather than development of bone. For these reasons Rb might not be a suitable candidate for targeted bone therapies.

#### p107 and p130

The proteins encoded by p107 and p130 are negative regulators of cell cycle. Hypo-phosphorylated forms of these proteins bind to the transcription factor E2F1 of p107 and p130, which leads to the G1 cell cycle to function in an antiproliferative stage [40, 64]. Cobrinik et al., demonstrated that p107 and p130 play a significant role in limb development by controlling the proliferation of chondrocytes [75]. Proliferative arrest of chondrocytes takes place when they differentiate into hypertrophic chondrocytes accompanied by loss of p107 and p130 [76]. p107 aids in induction of alkaline phosphatase encoding gene Alpl through recruitment of SWI/SNF chromatin remodeling complex [77]. p107 and p130 do present as potential targets to improve bone healing. However, this would require further research into teasing out the role of Rb from p107 and 130 as they form an intricate network.

## Conclusion

This review has comprehensively explored the role of cell cycle regulators in bone related processes like development and healing. Preclinical models and in vitro experiments have conclusively illustrated their intricate role in various stages of bone processes—MSC recruitment, differentiation to osteo/chondro progenitors, differentiation to bone or cartilage cells and proliferation of bone or cartilage cells. Of particular interest are CKI’s such as p21 and p27 that have demonstrated a direct role in bone healing. Future studies can be initiated on localized manipulation of theses regulators in specific cell types to improve bone healing while also preventing non-specific interactions. Several small molecules have been discovered targeting specific cell cycle regulators with respect to cancer that could be leveraged to bone healing applications. These findings have immense significance in discovering novel therapies to treat large bone fractures and improve outcomes in pathologies like osteoporosis or age related bone degeneration. After thorough investigation of these CKIs, delivery modes of gene manipulation will need to be undertaken such as microparticle mediated delivery, siRNA delivery etc. The targeting of cell cycle regulators in bone healing will serve as a paradigm shift in treatment strategies.

## Data Availability

Data shared here is available upon request.
